# Soil Chemical Properties, Metabolome, and Metabarcoding Give the New Insights into the Soil Transforming Process of Fairy Ring Fungi *Leucocalocybe mongolica*

**DOI:** 10.3390/jof8070680

**Published:** 2022-06-28

**Authors:** Mingzheng Duan, Meiling Lu, Jia Lu, Wenjing Yang, Bo Li, Li Ma, Lingqiang Wang

**Affiliations:** State Key Laboratory for Conservation and Utilization of Subtropical Agro-Bioresources, College of Agriculture, Guangxi University, 100 Daxue Rd., Nanning 530004, China; duanmingzheng@gxu.edu.cn (M.D.); 1931200417@st.gxu.edu.cn (M.L.); 1931200420@st.gxu.edu.cn (J.L.); 1931200203@st.gxu.edu.cn (W.Y.); 2117301021@st.gxu.edu.cn (B.L.); 2017301029@st.gxu.edu.cn (L.M.)

**Keywords:** fairy ring, *Leucocalocybe mongolica*, microbial fertilizer, metals, 16s rRNA, ITS, soil carbohydrate

## Abstract

A unique ecological landscape distributed in the Mongolian Plateau, called fairy rings, caused by the growth of the fungus *Leucocalocybe mongolica* (LM) in the soil could promote plant growth without fertilization. Therefore, this landscape can alleviate fertilizer use and has excellent value for agricultural production. The previous studies only investigated several parameters of the fairy rings, such as soil microbial diversity and some soil chemical properties, thus conclusions based on the studies on fairy rings lack comprehension. Therefore, the present study systematically investigated the chemical properties, metabolome, and metabarcoding of LM-transformed soil. We analyzed fairy ring soils from DARK (FR) and OUT (CK) zone correlated growth promotion with ten soil chemical properties, including N, nitrate-N, inorganic-P, cellulose, available boron, available sulfur, Fe, Mn, Zn, and Cu, which were identified as important markers to screen fairy ring landscapes. Metabolomics showed that the accumulation of 17 carbohydrate-dominated metabolites was closely associated with plant growth promotion. Finally, metabarcoding detected fungi as the main components affecting soil conversion. Among the various fungi at the family level, Lasiosphaeriaceae, unidentified_Auriculariales_sp, and Herpotrichiellaceae were markers to screen fairy ring. Our study is novel and systematically reveals the fairy ring soil ecology and lists the key factors promoting plant growth. These findings lay a theoretical foundation for developing the fairy ring landscape in an agricultural system.

## 1. Introduction

Excessive fertilization is a major problem in agricultural production, inhibiting crop productivity, polluting the environment, and threatening soil biodiversity in China and other developing countries [1,2,3,4]. Therefore, alternative methods to promote more efficient use of nutrients in soil to balance food production and environmental goals urgently need to be identified. One of the major approaches is to combine ecological phenomena with agricultural management to save energy and resources, then increase production. Fairy rings are one such natural phenomena which use ecological characteristics to promote efficient utilization of plant fertility. It is caused by the interaction between fungi and soil and has complex ecological processes that combine soil microbial diversity with metabolism, and chemistry properties with plant soil interaction [5,6,7].

**Figure 1:**
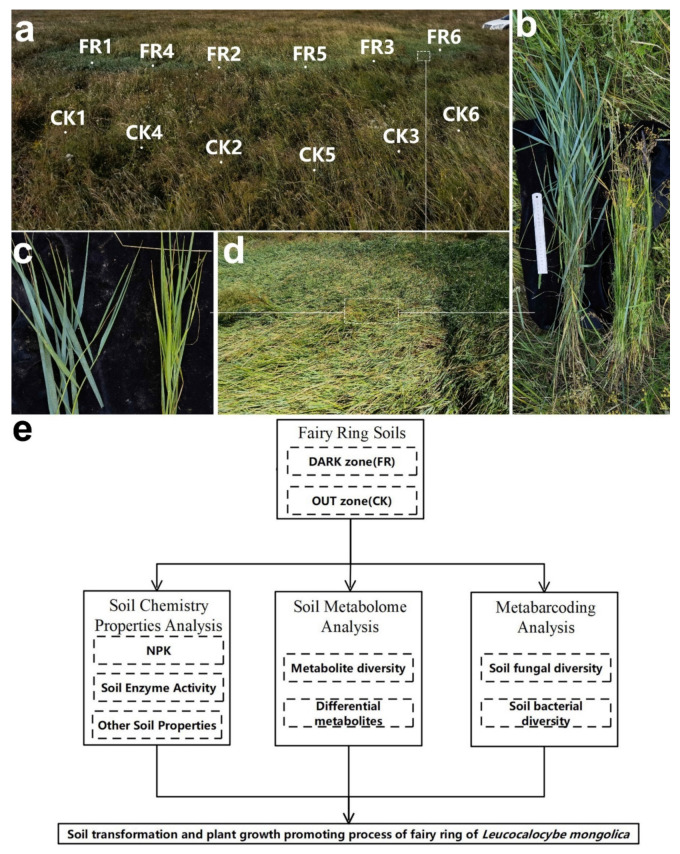
Apparent morphology of fairy rings and technical route of the study methods. (**a**) Fairy ring landscape and soil sampling point in this study, FR1-6 mean sampling point in DARK zone; CK 1-6 sampling point in OUT zone. (**b**) Comparison of plant height between DARK (**upper**) and OUT (**lower**) zones. (**c**) Comparison of leaf color between DARK (**left**) and OUT (**right**) zone; (**d**) Lush vegetation inside the DARK zone. (**e**) The technical route of this study.

Through our years of field survey of various fairy rings caused by different fungi. The fairy rings dominated by *Leucocalocybe mongolica* (LM, also known as a wild edible mushroom) are found distributed in the Inner Mongolia steppe region of China and have the ability to increase pasture (*Leymus chinensis*) production without fertilization (Figure 1). Plant growth promotion is concentrated in the DARK zone of the fairy rings [5]. The phenomenon is characterized mainly by the change in leaf color to dark green and the increased biomass in the DARK zone compared with outside. However, the mechanisms underlying growth promotion by fairy rings are unknown. Understanding these will help us artificially develop the ecological landscape and solve the problem of excessive fertilization in agricultural production.

Generally, fairy rings are formed due to an interaction between plants and soil fungi, such as *Marasmius oreades* [8] and *Lepista sordida* [9], and are divided into three types based on an early study [10]. Among them, Type I fairy rings have stronger soil interaction ability, and therefore have stronger development potential, and LM belong to Type I [5]. Most fairy ring fungi can live in a variety of habitats. *Lepista sordida* is found distributed in forests, farmlands, and grasslands. Meanwhile, LM is of lake habitat adaptation only and could only live in fairy ring habitats due to the contraction of its gene family [11]. Researchers believe that the fairy ring fungi influence soil fertility and transform the soil, which manipulates the plants and soil microbes and results in a unique ecosystem. Studies have shown that fairy rings could increase plant biomass and regulate plant diversity [6,12], influence soil microbial diversity [13,14], and improve soil physical and chemical properties [15,16]. Fairy ring fungi could be found to secrete metabolites, such as imidazole-4-carboxamide and 2-azahypoxanthine, which promote the growth of plants by stimulated plant root growth [7,17,18,19]. These earlier findings have indicated the ecological value of fairy ring landscapes and fairy ring fungi. However, the above studies are independent and lack a detection range, and no studies have systematically explored the ecology of the same type of fairy ring, including those that have discovered various soil chemical properties, microbial diversity, and metabolomes within the same study.

Meanwhile, the progress in soil metabolomics in recent years has correlated new fingerprint types to several environmental factors, including soil nutrients, microbial diversity, and plant phenotypes [20]. Song found that crop species, plant genotypes, and growth substrates influence soil metabolomics [21]. In addition, soil metabolomes can be regulated by soil microbes and soil chemistry properties, e.g., the wave of soil carbohydrate metabolites can be reflected in the status of soil organic matter and microbial activity [22,23]. Therefore, in our study, we introduce a soil metabolomic evaluation that will provide new insights into the soil ecology of fairy rings.

Several studies have reported the impact of fairy rings on soil microbes, plants, and soil physical and chemical properties; however, the outcomes of the results of these studies are lacking comprehension. In studies on several different fairy rings soils, the results only include soil microbial diversity and some soil chemical properties, and yet no studies of enzyme activity and metabolome in fairy ring soils have been reported [5,6,12,13]. Therefore, systematic and in-depth research on fairy rings is required to identify and select a model species for landscape development.

Therefore, in order to reveal the transformation process of soil ecology during LM growth, the promotion of plants in the FR zone of a fairy ring is essential. The present study systematically analyzed the soil chemistry, soil metabolism, and soil microbial diversity in a fairy ring ecosystem with an obvious plant growth promoting function (LM). The study investigated the changes in multidimensional factors during soil conversion by fairy rings under the same environmental background. We reveal a number of potential factors that may contribute to the growth promoters of LM fairy rings.

## 2. Materials and Methods

### 2.1. Materials

The soil samples were collected from an LM fairy ring landscape with remarkable plant growth-promoting properties (Figure 1a) located in a prairie in Baorixile Town, Hulun Buir City, Inner Mongolia autonomous region, China (49°72′ N, 119°54′ E, and an elevation of 694 m). In this landscape, the plant height (Figure 1b), leaf color (Figure 1c), and plant density and biomass (Figure 1d) were significantly high in the DARK and OUT zones. The pH of the sampled soil was 6.4 ± 0.2. The sampling area was divided into DARK and OUT zones based on our earlier description [5]. Six sampling sites (duplicates) were uniformly selected in the shape of an arc, including the fairy ring’s DARK zone (FR) and the OUT zone (CK). As Figure 1d shows, the sampling site CK1–3 and FR1–3 samples were used for all analyses, while CK4–6 and FR4–6 were used as the biological replicates for metabolomics. For each sampling spot, the soil was collected from the top layer using a soil sample collector (depth, 0–10 cm; diameter, 5 cm), sieved (0.425 mm filter), packed into sterile cryo-storage tubes, and frozen in liquid nitrogen [24].

### 2.2. Methods

We used the soil chemistry, metabolome, and metabarcoding results to elucidate how fairy ring fungi alter the soil in the natural environment (Figure 1e).

#### 2.2.1. Assessment of Soil Chemical Properties

We analyzed 38 chemical properties of the soil samples following the “National Standards of the People’s Republic of China” and using the standard kits. Refer to Supplementary File S1 for details. Welch’s *t*-tests were performed by GraphPad Prism software (v 9.3.0) in our study to evaluate the results.

#### 2.2.2. Soil Metabolome Survey

Approximately 0.5 g of soil sample was mixed with 1 mL of methanol: isopropanol: water (3:3:2 V/V/V) mixture, vortexed for 3 min, and placed on an ultrasound for 20 min. The extract was centrifuged at 12,000 r/min at 4 °C for 3 min, and the supernatant was mixed with 0.020 mL internal standard (10 μg/mL) in a sample vial and allowed to evaporate under nitrogen flow. The sample was freeze-dried in a lyophilizer, and the residue was used for derivatization. The sample was mixed with 0.1 mL of methoxyamine hydrochloride in pyridine (0.015 g/mL) and incubated at 37 °C for 2 h. Then, 0.1 mL of Bis(trimethylsilyl)trifluoroacetamide/BSTFA (with 1% Trimethylchlorosilane/TMCS) was added into the mixture and kept at 37 °C for 30 min after vortex-mixing. Approximately 0.2 mL of the derivatization solution was mixed with n-hexane to dilute it to 1 mL, filtered through a 0.22 μm organic phase syringe filter, stored in a refrigerator at −20 °C, and analyzed within 24 h. The metabolites in the soil samples were analyzed by gas chromatography-mass spectrometry (GC-MS) on an Agilent 8890 gas chromatography coupled to a 5977B mass spectrometer with a DB-5MS column (30 m length × 0.25 mm i.d. × 0.25 μm film thickness, J&W Scientific, Palo Alto, CA, USA). Helium was used as the carrier gas at a 1.2 mL/min flow rate. Samples were injected in the front inlet mode with a split ratio of 5:1, and the injection volume was set to 1 μL. The oven temperature was held at 40 °C for 1 min and then raised to 100 °C at 20 °C/min, raised to 300 °C at 15 °C/min, and held at 300 °C for 5 min. All samples were analyzed in scan mode. The ion source and transfer line temperature were set at 230 °C and 280 °C, respectively.

In statistics, unsupervised PCA (principal component analysis) was performed using the statistics function prcomp within R (www.r-project.org, accessed on 20 January 2022), and unit variance-scaling was applied to the data before unsupervised PCA. Metabolites with a VIP ≥ 1 and an absolute log2FC (fold change) ≥ 1 were identified as significantly different between the groups of CK (CK1-6) and FR (FR1-6). We used VIP (Variable important in projection) values to identify differential metabolites, and VIP values were extracted from the orthogonal partial least squares discriminant analysis (OPLS-DA) results, containing score plots and permutation plots generated using the R package MetaboAnalystR. Finally, the original data of this study were log2-transformed and mean-centered before OPLS-DA. A permutation test (200 permutations) was performed to avoid overfitting.

#### 2.2.3. Metabarcoding Survey

As described previously [5], DNA was extracted from 3 g of soil sample using the HiPure Soil DNA Kit (Magen, Guangzhou, China) according to the manufacturer’s protocol. The 16S rDNA V3-V4 region of the ribosomal RNA gene was amplified by PCR using the 341F (CCTACGGGNGGCWGCAG) and 806R (GGACTACHVGGGTATCTAAT) primers for bacteria [25] and the ITS3_KYO2 (GATGAAGAACGYAGYRAA) and ITS4 (TCCTCCGCTTATTGATATGC) primers for fungi [26]. The purified amplicons were pooled in equimolar ratios and paired-end sequenced (PE250) on an Illumina platform (Novaseq 6000 sequencing) according to the standard protocol. Representative operational taxonomic unit (OTU) sequences were classified through a naïve Bayesian model using an RDP classifier [27] (version 2.2) based on the SILVA database (version 132) for bacterial taxonomy (16S rRNA metabarcoding data) [28] and the UNITE database (version 8.0) for fungal taxonomy (ITS metabarcoding data) [29], with a confidence threshold value of 0.8. All figures were generated using R projects. Venn analysis to compare the OTU among the different groups was performed in R using the VennDiagram package (version 1.6.16) [30]. Alpha diversity was analyzed by calculating Sob (assessed species richness level) and Shannon and Chao1 (comprehensively assessed richness and evenness of species) indices in QIIME [31] (version 1.9.1). Finally, a principal component analysis (PCA) was performed in R using the vegan package (version 2.5.3; http://CRAN.R-project.org/package=vegan; accessed on 9 August 2021) to assess the sample composition relation. All analyses in this section were performed using the numbers of OTU without any model transformation. LEfSe (linear discriminant analysis effect size) analysis was performed using the LEfSe software [32], and the value of LDA filtrate score was 4.

## 3. Results and Discussion

### 3.1. Soil Chemical Properties

#### 3.1.1. Soil NPK Status

Firstly, we analyzed the nitrogen (N), phosphorus (P) and potassium (K) in soils from CK and FR zones. We analyzed 13 soil chemical properties, including N (2703 mg/kg to 3303 mg/kg), nitrate-N (11.47 mg/kg to 18.34 mg/kg), nitrite-N (0.39 mg/kg to 0.48 mg/kg), alkaline-N (198 mg/kg to 234 mg/kg), ammonia-N (14.93 mg/kg to 18.42 mg/kg), micro-N (35.03 mg/kg to 84.45 mg/kg), P (432 mg/kg to 474 mg/kg), organo-P (361 mg/kg −398 mg/kg), inorganic-P (51.75 mg/kg to 71.31 mg/kg), alkaline-available P (2.9 mg/kg to 6.28 mg/kg), K (16,918 mg/kg to 18,416 mg/kg), available-K (317 mg/kg to 438 mg/kg), and slowly available-K (3333 mg/kg to 3987 mg/kg) (Table 1); most of the properties were significantly different between the soils of CK and FR zones. Nitrite-N (*p* < 1 × 10^−4^), ammonia-N (*p* = 0.0048), micro-N (*p* = 0.0005), alkaline-available P (*p* = 0.0003), alkaline-available P (*p* = 0.0003), available-K (*p* < 1 × 10^−4^), and slowly available-K (*p* = 0.0014) were significantly higher in CK than in FR, while N (*p* = 0.021), nitrate-N (<1 × 10^−4^), and inorganic-P (0.016) were higher in FR than in CK. Meanwhile, the differences in the remaining properties, including alkaline-N, P, organo-P, and K, were not obvious. These observations indicate that the soil nutrient status of FR was poorer than CK. Studies on fairy ring soil of the genus *Agaricus* showed that the total N and P were lower in the FR zones than in CK [12], consistent with our results. However, nitrite-N and available P were higher in FR [33], which is different from our results, probably due to environmental differences. This suggests that the background of FR ecology is complex, so we propose that studies on the ecology of the fairy ring should try to select a fixed geographical location and specific fairy ring fungi for development. Interestingly, both studies indicate that nitrate-N is an important factor affecting plant growth promotion in a fairy ring ecosystem. Nitrate-N is the most major component of nitrogen fertilizer in agricultural practice [34], and the main forms are taken up by plants [35], which partly explained the reason why the plant in the FR zone should be promoted. Finally, based on the above, fairy rings show the function of improving soil nutrients, especially in nitrogen related elements (N and nitrate-N), thus we believe that the LM fairy ring is an ecological phenomenon with soil nitrogen fixation transformation ability, and its nitrogen fixation process is worth exploring in the following research.

#### 3.1.2. Soil Enzyme Activity

Soil enzymes serve as one of the most active organic components in the soil and are involved in various soil biochemical processes and assist in plant growth [36]. Therefore, we examined the activity of nine soil enzymes (Table 2), only cellulase (higher in FR than in CK, *p* = 0.0002, range at 1790 mg/kg to 2816 mg/kg), urease (higher in CK than in FR, *p* = 0.0005, range at 281 mg/kg to 414 mg/kg), and sucrase (higher in CK than in FR, *p* = 0.02, range at 117 mg/kg to 127 mg/kg) were significantly different between FR and CK. Meanwhile, the concentration of only cellulase, urease, sucrose, and β-glucosidase exceeded 50 mg/kg, which may be one of the main influencing factors. However, the activity of cellulose alone was overwhelmingly dominant in FR, which suggests its role in plant growth promotion. Cellulase is an important enzyme complex that hydrolyzes cellulose to oligosaccharides and glucose [37]. Soil fungi and bacteria are the sources of cellulase [38,39]. Its high activity may be related to the increase of soil nitrogen content in the FR zone [40]. We suppose that the high plant biomass in the FR zone also provides an additional source of plant fiber in the soil, providing conditions for cellulase synthesis. Consequently, it has a positive feedback on the soil and plant growth again. Taken together, we found that the fairy ring has an ecological function to regulate soil cellulase content, which is a very important soil improvement property and deserves further verification and application in soil improvement research.

#### 3.1.3. Other Soil Properties

We also analyzed 16 other soil chemical properties influencing plant growth (Table 3), including organic matter (63,213 mg/kg to 68,480 mg/kg), carbon (105,300 mg/kg to 123,700 mg/kg), dissolved organic carbon (37.32 mg/kg to 53.02 mg/kg), micro-carbon (390 mg/kg to 591 mg/kg), boron (18.31 mg/kg to 28.15 mg/kg), available boron (0.48 mg/kg to 1.03 mg/kg), sulfur (104 mg/kg to 122 mg/kg), available sulfur (9.24 mg/kg to 11.74 mg/kg), salt (2688 mg/kg to 3424 mg/kg), manganese (Mn; 11.18 mg/kg to 41.3 mg/kg), iron (Fe; 20.57 mg/kg to 241.43 mg/kg), copper (Cu; 0.56 mg/kg to 2.67 mg/kg), zinc (Zn; 1.65 mg/kg to 81.47 mg/kg), sodium (Na; 12,930 mg/kg to 17,821 mg/kg), magnesium (Mg; 4993 mg/kg to 5783 mg/kg), and calcium (Ca; 5208 mg/kg to 6180 mg/kg). Interestingly, we found that the metal ions Mn (*p* = 0.02), Fe (*p* < 1 × 10^−4^), Zn (*p* < 1 × 10^−4^), and Cu (*p* < 1 × 10^−4^) were significantly higher in FR than in CK, which may suggest the metal ion enrichment capability of LM in the fairy rings. Earlier, Gramss found that the DARK zone of the fairy ring of *Marasmius oreades* was highly enriched with Fe [16], and Zotti also found that the DEAD zone of the fairy ring of *Calocybe gambosa* was highly enriched with Fe [41]. Mushrooms typically have a strong ability to enrich metals [42]. In the fairy ring ecosystem, they may bring trace metals that are missing from CK to FR soil. During the plant growth process, Fe and Cu are most often used for their redox properties, whereas Zn is primarily used for its ability to act as a Lewis acid [43]. These trace metals, particularly Fe, promoted plant growth in the FR zone. Moreover, the enriched Fe probably resulted in the dark green leaves in the FR zone. In a study on *Areca catechu*, an excess of Fe caused the leaves to turn dark green [44], which justifies the dark green leaves in the FR zone enriched with Fe. In our study, the multipurpose trace metal Zn was also highly enriched in FR, confirming the close relationship between a growth-promoting feature of fairy rings and metal ions.

Therefore, we believe that trace metals are essential for plant growth promotion by fairy rings and should be further explored. In summary, the presented results demonstrate that fairy rings have the potential ecological function of soil metal adsorption, which may be of great significance for soil improvement studies, because it can be used not only to help improve soil types lacking in metal elements, but also to improve soils with heavy metal pollution.

Furthermore, we found that various other chemical properties, including organic matter (*p* = 0.0005), dissolved organic carbon (0.007), micro-carbon (0.001), and Na (0.01), were significantly higher in CK than in FR, while available boron (0.0003) and available sulfur (0.009) were significantly higher in FR. Boron and sulfur influence plant growth [45,46], therefore, it is likely to be involved in plant growth promotion in fairy rings. Interestingly, Na, an unwanted and growth-inhibiting element, was downregulated in the FR zone, indicating that LM plays a role in regulating soil salinity; however, this deserves further verification.

### 3.2. Soil Metabolome

#### 3.2.1. Metabolite Composition

**Figure 2:**
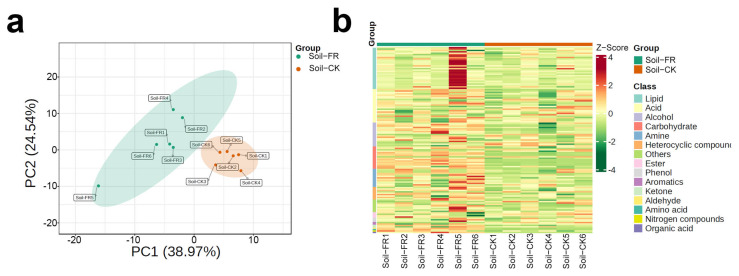
Distribution of soil metabolites in the FR and CK zones of the fairy ring ecosystem. (**a**) Principal component analysis (PCA) score plots of soil metabolome. PC1 represents the first principal component, and PC2 represents the second principal component. Percentage values indicate the proportion of variance explained. (**b**) Heat map clustering of the soil metabolites. Sample names are presented along the horizontal axis, and metabolite classes are written vertically; different colors on the map indicate the relative content of the metabolite (red represents high content, green represents low content).

Soil metabolomics could correlate fingerprint type to environmental factors, soil nutrients, microbial diversity, and plant phenotypes [20]. Thus, we performed the first soil metabolomics study in the FR and CK zones to form a fairy ring ecology. Soil metabolome studies usually have a large margin of error; therefore, we maintained six biological replicates per treatment to improve credibility. The PCA plot showed that the samples in the FR zone were significantly different from those in the CK zone and were clustered into two separate groups (Figure 2a). Metabolomic analysis of the soils from 12 sites (Figure 1d) in the two zones showed that the metabolite composition of the FR zone was different from the CK zone. Based on the z-scores (Standardized Population Data), we evaluated the categories and abundance of metabolites. As shown in Figure 2b, 134 metabolites grouped into 15 classes were detected in all soils, including acids (25 of 134), alcohols (17), aldehydes (1), amines (11), amino acids (1), aromatics (2), carbohydrates (16), esters (4), heterocyclic compounds (9), ketones (2), lipids (30), nitrogen compounds (1), organic acids (1), phenols (3), and others (9). Earlier studies have proven that carbohydrates and organic acids are essential for plant growth and usually dominate soils, and these compounds are generally derived from the root exudates of plants [47]. In this study, lipids and acids were identified as the most diverse metabolite classes in all samples, and carbohydrates were shown to be the most enriched in the FR group (Figure 2b). Carbohydrates are a significant component of the organic matter of all soils, commonly accounting for 5–20% of soil organic matter [48], and it can be can be an indicator of soil physical quality [49], thus suggesting that fairy rings may improve soil quality by regulation of soil metabolome. Moreover, we found that the enrichment of carbohydrates in the FR zone is associated with the trend observed for cellulase (Section 3.1.2), suggesting that cellulase enrichment causes carbohydrate enrichment in the FR group. Consequently, more carbohydrates are available for plant growth promotion in the fairy rings than in control. However, the link between carbohydrate enrichment and plant growth promotes the need for further investigation. Finally, we provide information on all 134 of the soil metabolites in Supplementary Table S1.

#### 3.2.2. Differential Metabolites

Furthermore, we identified 17 metabolites significantly different between the FR and CK soils (VIP > 1.5 and *p*-value < 0.05). Detailed analysis showed that 10 of 17 belonged to the carbohydrate class (Table 4); among these, eight differential carbohydrates, including sucrose, galacto-heptulose 2, D(+)-talose, fructose 2, D-allose 2, rhamnose, D-arabinitol 2, and 1-deoxy-d-arabitol, showed higher expression in the FR zone than in the CK zone. In soils, carbohydrates contribute significantly to carbon stock and aggregate stability, and can also be used to estimate changes in soil organic matter [22]. Approximately 80% of soil carbohydrates are derived from soil microbes [23], so the result implies that, in the present study, soil microbes are more active in the FR than in the CK zone, therefore the accumulation of carbohydrates in the FR zone may justify the plant growth promotion in the fairy rings. In addition, 22-hydroxydocosanoic acid (acid), 2,3,4-trihydroxy-3-(hydroxymethyl)butanal (aldehyde), N-tetradecyl-2-butylamine (amine), 5-oxo-DL-proline (amino acid), and malic acid (organic acid) among the upregulated metabolites of the FR soil may also be involved in FR plant growth promotion. Therefore, we identified 17 signature carbohydrate-dominated metabolites of fairy ring soil, possibly promoting plant growth character, and they need detailed analysis in in vitro studies. In addition, for fairy ring metabolites found in *Lepista sordida* [7,17,18,19], imidazole-4-carboxamide and 2-azahypoxanthine, these two metabolites or their related metabolites were not detected in our results. This may be due to differences in the fairy ring fungi, or to the fact that the plant had already consumed them at the time we sampled soils. Further analysis of the root metabolome and comparison with the soil metabolome will help determine the source and role of these metabolites.

### 3.3. Soil Microbial Diversity

#### 3.3.1. Metabarcoding

**Figure 3:**
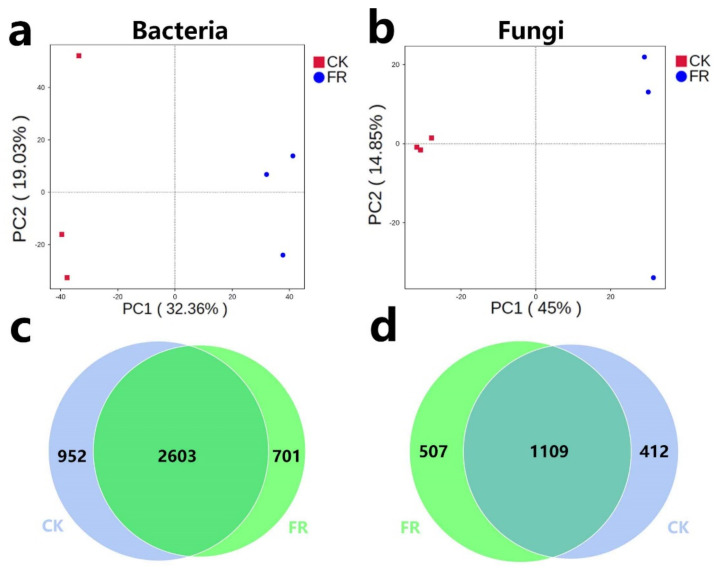
PCA and Venn analysis of bacteria and fungi in the FR and CK zones of the fairy ring ecosystem. (**a**,**b**) PCA of the bacterial and fungal OTUs in the FR and CK soils. The colored dots in the figures represent the different sample groups. (**c**,**d**) Venn analysis of the bacterial and fungal OTUs.

Soil microbial diversity can be regulated by fairy rings [5,6]. To explore the diversity of soil fungi and bacteria in plant growth promotion process in the FR and CK zones, we performed metabarcoding survey. A total of 866,128 effective metabarcoding tags were obtained via 16S rRNA and ITS sequencing. The OTU clustering of the soil samples identified 2582 bacterial OTUs (16S rRNA) and 1195 fungal OTUs (ITS) on an average per sample (Table 5). PCA plots based on OTU abundance showed that fungi and bacteria could separate clusters by the CK and FR groups, which means their microbial distributions were significantly different (Figure 3a,b). The VENN diagram of the OTUs showed that 2603/4256 (61.16%) of the bacterial OTUs and 1109/2028 (54.68%) of the fungal OTUs were common across all soils, while 701/4256 (16.47%) of the bacterial OTUs and 507/2028 (25%) of the fungal OTUs were unique to the FR zone (Figure 3c,d). OTU diversity observed in this study is similar to our previous results on the LM fairy rings [5,24]. However, compared with the other study results of fairy ring soils, the OTU numbers were lower than LM; for example, only 1242 to 1398 bacterial OTUs were detected in the fairy ring soils of *Agaricus gennadii*, which was much lower than our results [12]. Our previous study also showed that the DARK zone soil had a genetic diversity advantage [5], while the present study showed that the DARK zone soil of the LM fairy ring had more microbial diversity than the fairy ring of other fungi.

#### 3.3.2. Soil Microbial Alpha Diversity

**Figure 4:**
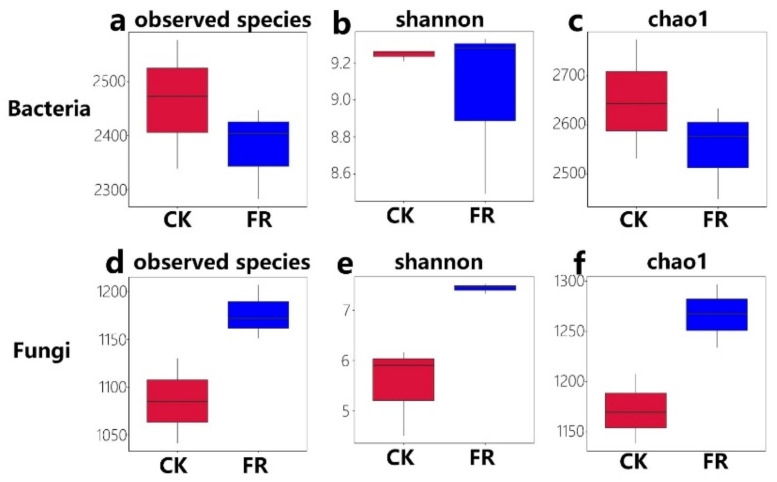
Alpha diversity of bacteria and fungi in the FR and CK zones of the fairy ring ecosystem. Boxes show: the top whisker represents the maximum value, the bottom whisker represents the minimum value, the line inside the box represents the median, the top margin of the box represents the upper quartile, and the lower margin of the box represents the lower quartile. (**a**,**d**) observed species indices of bacteria and fungi; (**b**,**e**) shannon indices of bacteria and fungi; (**c**,**f**) chao1 indices of bacteria and fungi.

Furthermore, we determined the alpha diversity and assessed the species diversity among the zones via a *t*-test. The values of the observed species (Sob) index of soil samples from the FR and CK groups ranged from 2283 to 2577 (Bacteria, Figure 4a) and 1041 to 1207 (Fungi, Figure 4b), respectively; the Shannon index ranged from 8.49 to 9.32 (Bacteria, Figure 4c) and 4.5 to 7.5 (Fungi, Figure 4d), respectively; and the Chao1 index ranged from 2448 to 2774 (Bacteria, Figure 4e) and 1138 to 1296 (Fungi, Figure 4f), respectively. Additionally, significant differences were detected in fungal diversity between the FR and CK zones (e.g., sob index, *p* = 0.04; Figure 4b; Chao1, *p* = 0.02; Figure 4c), but less difference was observed in bacterial diversity. Compared to our earlier study on LM fairy rings, the stability of soil fungal diversity was higher than that of bacteria. The previous study showed that the bacterial sob index ranged from 3694 to 4255, but the fungal sob ranged from 1009 to 1325 [5], which was quite different from the present study. The present results indicate that fungi have a stronger influence than bacteria on the ecology of fairy rings of LM.

#### 3.3.3. Key Soil Microbial Taxa

**Figure 5:**
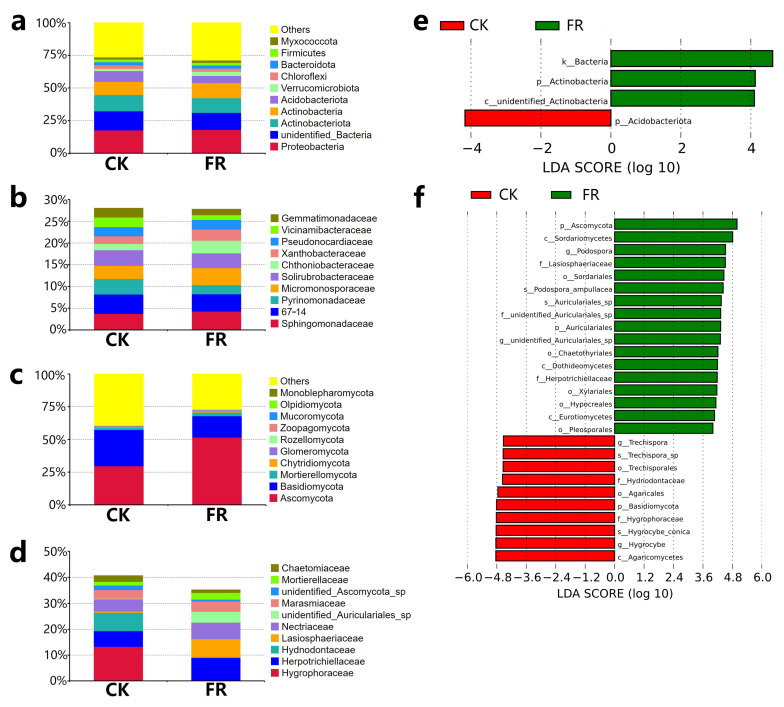
Microbial community composition and LEfSe analysis of bacteria and fungi in the FR and CK zones of the fairy ring ecosystem. The relative abundance of the bacteria community at the (**a**) phylum level and (**b**) family level. The relative abundance of the fungal community at the (**c**) phylum level and (**d**) family level. LEfSe analysis of (**e**) bacteria and (**f**) fungi.

Furthermore, we analyzed differences in the bacterial and fungal community compositions between the FR and CK zones based on the SILVA and UNITE databases. The microbial taxa detected are shown in Figure 5. The analysis revealed that the top five most abundant bacterial phyla (Figure 5a) were Proteobacteria (17.97% and 17.61% in FR and CK), unidentified_Bacteria (13.04% and 14.65%), Actinobacteriota (11.26% and 12.51%), Actinobacteria (11.67% and 10.09%), and Acidobacteriota (5.41% and 8.21%). While the top five bacterial families (Figure 5b) were Sphingomonadaceae (4.29% and 3.73%), 67–14 (3.95% and 4.45%), Pyrinomonadaceae (2.09% and 3.57%), Micromonosporaceae (3.91% and 9.04%), Solirubrobacteraceae (3.39% and 3.56%). The top three most abundant fungal phyla (Figure 5c) were Ascomycota (42.92% and 37.3% in FR and CK), Basidiomycota (16.29% and 27.71%), and Mortierellomycota (2.7% and 1.8%). The top five most abundant fungal families (Figure 5d) were Hygrophoraceae (0.16% and 13.26%), Herpotrichiellaceae (8.8% and 6.08%), Hydnodontaceae (0.11% and 7.06%), Lasiosphaeriaceae (7.16% and 0.52%), and Nectriaceae (6.38% and 4.66%). LEfSe analysis (Figure 5e,f) identified Actinobacteria (log_10_ LDA score > 3, *p* < 0.05) as the only marker bacterial phylum in FR zone, and three fungal families, Lasiosphaeriaceae, unidentified_Auriculariales_sp, and Herpotrichiellaceae (log 10 LDA score > 3, *p* < 0.05) as marker units in FR zone. These observations suggest that the ecology of the fairy rings does not interfere with the distribution of bacteria but could strongly affect the fungi, consistent with our previous conclusion [5,24]. This suggests that fungi are the focus of the isolation of plant growth-promoting microbes. Combining the above results of differential metabolites and soil chemical properties, these key soil microbial taxa may be associated with them. E.g., these fungi families may participate in processes of nitrification/denitrification in soil, that could transform nitrate-N for plants in fairy ring.

Among the marker bacteria and fungi associated with the FR zone, Actinobacteria is known to improve the availability of nutrients and minerals, enhance the production of metabolites, and promote plant growth regulators [50]; thus, its enrichment may promote the growth of plants in the FR zone of the fairy ring. Meanwhile, Lasiosphaeriaceae found enriched in the FR may have the ability to regulate soil metal ion enrichment. Li et al. showed Lasiosphaeriaceae regulates soil aluminum in a study conducted on wheat [44]. In the role of Herpotrichiellaceae in soil, Cui et al. significantly correlated Herpotrichiellaceae with microbial N limitation in the desert-grassland ecological transition zone [51], may indicating the role of in regulating the soil nitrogen cycle in the FR zone. In addition, Nectriaceae was found enriched in the FR zone, consistent with our previous studies [5]; however, the LEfSe analysis did not support it as a marker family. A study showed that Nectriaceae might regulate soil salinity [52]. The present study preliminarily evaluated that in LM fairy ring ecology, bacterial diversity may be less correlated with plant growth promotion characteristics, while fungi may play a major role in the direct correlation between soil microbes and the plant growth promotion of characteristics of fairy rings.

## 4. Conclusions

The present study is the most systematic study on the fairy ring soil ecology at present. We investigated the fairy ring landscape’s soil chemical properties, metabolome, and microbes. Our detailed analysis showed that soil carbohydrates, metal ions, and a few fungi are the key factors promoting plant growth in the DARK zone of fairy rings. Our study has defined the scope for revealing the mechanisms of the fairy ring that ecologically promote plant growth, which lays the foundation for developing fairy ring ecology and provides theoretical information for further research.

## Figures and Tables

**Table 1:** Nutrient status (NPK) of the soils in FR compared with CK zones in a fairy ring ecosystem. The numbers mean mg/kg.

ID	CK			FR			*p* Value	Type
	CK1	CK2	CK3	FR1	FR2	FR3		
N	2795.83	2703.68	2825.42	2909.43	3033.36	2951.51	0.0213	upregulated
nitrate-N	11.48	12.11	12.36	18.34	17.52	17.83	<0.0001	upregulated
nitrite-N	0.3987	0.3960	0.3983	0.0469	0.0488	0.0475	<0.0001	downregulated
alkaline-N	219.29	218.10	198.92	234.87	229.87	216.54	0.1552	non-significant
ammonia-N	18.43	17.39	17.60	15.57	14.94	15.68	0.0036	downregulated
micro-N	591.88	565.30	541.23	404.29	420.53	390.30	<0.0001	downregulated
P	471.91	457.57	474.98	459.12	450.56	432.82	0.0935	non-significant
organo-P	390.14	398.88	394.07	384.34	372.05	361.37	0.0374	downregulated
inorganic-P	52.72	55.23	51.76	65.17	71.31	63.61	0.0063	downregulated
alkaline available-P	6.28	6.04	6.26	2.90	3.04	2.53	<0.0001	downregulated
K	18,161.25	17,639.43	18,416.79	16,918.35	17,968.72	17,108.40	0.1347	non-significant
available-K	438.25	435.59	426.41	317.71	319.99	325.18	<0.0001	downregulated
slowly available-K	3987.07	3908.38	3903.42	3333.36	3352.78	3337.52	<0.0001	downregulated

**Table 2:** Enzyme activities status of soils in FR compared with CK zones in a fairy ring ecosystem. The numbers mean mg/kg.

ID	CK			FR			*p* Value	Type
	CK1	CK2	CK3	FR1	FR2	FR3		
cellulase	1799.58	1806.84	1790.85	2816.05	2775.05	2747.78	0.0002	upregulated
urease	399.98	414.09	408.61	285.08	282.83	281.26	0.0005	downregulated
sucrase	123.04	127.48	126.95	120.20	120.46	117.19	0.0231	downregulated
catalase	3.27	3.10	3.11	2.90	3.08	2.89	0.0687	non-significant
dehydrogenase	0.0082	0.0063	0.0066	0.0063	0.0078	0.0086	0.5728	non-significant
β-glucosidase	208.95	276.29	228.90	244.36	210.90	232.91	0.724	non-significant
acid phosphatase	1.85	1.76	1.69	1.86	2.07	1.99	0.0594	non-significant
neutral phosphatase	1.39	1.38	1.67	1.46	1.68	1.53	0.5358	non-significant
alkaline phosphatase	1.77	1.71	1.74	1.41	1.64	1.36	0.0883	non-significant

**Table 3:** Other properties status of soils in FR compared with CK zones in a fairy ring ecosystem. The numbers mean mg/kg.

ID	CK			FR			*p* Value	Type
	CK1	CK2	CK3	FR1	FR2	FR3		
organic matter	68,379	68,129	68,480	63,213	63,842	63,946	0.0005	downregulated
carbon	119,500	117,300	121,400	123,700	105,600	105,300	0.3244	non-significant
dissolved organic carbon	50.15	49.41	53.03	42.69	37.32	38.06	0.0071	downregulated
micro-carbon	591.88	565.30	541.23	404.29	420.53	390.30	0.0018	downregulated
boron	19.07	28.16	25.33	24.17	28.06	18.31	0.8716	non-significant
available boron	0.5584	0.5665	0.4804	0.9437	1.0321	0.9958	0.0003	downregulated
sulfur	104.84	112.24	112.76	112.18	122.74	117.89	0.1292	non-significant
available sulfur	9.25	9.76	10.11	11.12	11.03	11.74	0.0094	upregulated
salt	3424.66	2939.88	3087.97	2791.18	2704.87	2688.49	0.0921	non-significant
manganese	11.70	11.18	11.44	39.39	41.30	29.05	0.022	upregulated
iron	20.58	22.54	29.12	231.58	241.35	224.84	<0.0001	upregulated
copper	0.56	0.68	0.56	2.68	2.67	2.49	<0.0001	upregulated
zinc	1.65	1.65	1.69	79.29	81.47	80.97	<0.0001	upregulated
sodium	17,515.31	17,821.64	15,953.07	14,136.30	14,455.60	12,930.58	0.0131	downregulated
magnesium	5163.16	5330.03	4993.51	5603.07	5783.24	5154.78	0.1935	non-significant
calcium	6180.07	5900.17	5869.81	5951.18	5887.98	5208.89	0.3362	non-significant

**Table 4:** Differential metabolites in the FR zone.

Compounds	Formula	Class	VIP	*p*-Value	Type
22-Hydroxydocosanoic Acid	C22H44O3	Acid	1.64	6.05 × 10^−8^	upregulated
Benzoic Acid	C7H6O2	Acid	1.65	1.30 × 10^−6^	downregulated
2,3,4-Trihydroxy-3-(Hydroxymethyl)Butanal	C5H10O5	Aldehyde	1.66	3.63 × 10^−6^	upregulated
N-tetradecyl-2-ButylAmine	C18H39N	Amine	1.67	9.41 × 10^−8^	upregulated
5-oxo-DL-Proline	C5H7NO3	Amino acid	1.64	3.15 × 10^−5^	upregulated
4-(1-methyl-1-phenylethyl)-Phenol	C15H16O	Aromatics	1.61	1.54 × 10^−4^	downregulated
Sucrose	C12H22O11	Carbohydrate	1.63	4.73 × 10^−4^	upregulated
.beta.-D-galactopyranosyl-4-O-.beta.-D-Glucopyranose 2	C12H22O11	Carbohydrate	1.56	2.09 × 10^−4^	downregulated
Galacto-heptulose 2	C7H14O7	Carbohydrate	1.66	5.65 × 10^−6^	upregulated
D(+)-Talose	C6H12O6	Carbohydrate	1.67	3.15 × 10^−4^	upregulated
Glucose 1	C6H12O6	Carbohydrate	1.61	2.63 × 10^−5^	downregulated
Fructose 2	C6H12O6	Carbohydrate	1.66	2.23 × 10^−4^	upregulated
D-Allose 2	C6H12O6	Carbohydrate	1.66	3.63 × 10^−4^	upregulated
Rhamnose	C6H12O5	Carbohydrate	1.66	6.73 × 10^−8^	upregulated
D-Arabinitol 2	C5H12O5	Carbohydrate	1.62	7.00 × 10^−4^	upregulated
1-Deoxy-d-arabitol	C5H12O4	Carbohydrate	1.62	1.33 × 10^−5^	upregulated
Malic Acid	C4H6O5	Organic acid	1.55	7.98 × 10^−3^	upregulated

**Table 5:** Bacterial and fungal metabarcoding sequencing data.

	Bacteria			Fungi		
Sample ID	Clean Tags	Average Length (nt)	OTU Number	Clean Tags	Average Length (nt)	OTU Number
CK1	63,849	400	2672	87,545	257	1164
CK2	64,458	404	2761	86,421	257	1197
CK3	53,402	417	2339	88,679	249	1128
FR1	64,249	400	2483	74,847	248	1207
FR2	66,006	409	2635	78,312	247	1215
FR3	55,966	405	2602	82,394	246	1259

## Data Availability

The raw amplicon sequencing dataset of metabarcoding is available in the NCBI Sequence Read Archive under BioSample accession PRJNA835297.

## Supplementary Materials

The following supporting information can be downloaded at: https://www.mdpi.com/article/10.3390/jof8070680/s1, Supplementary File S1: Methods of assessment of soil chemical properties [53,54,55,56,57,58,59,60,61,62,63,64,65,66,67,68,69,70,71,72,73]; Table S1: Information of 134 soil metabolites form soils.
