# Supplementary material for: Soil Chemical Properties, Metabolome, and Metabarcoding Give the New Insights into the Soil Transforming Process of Fairy Ring Fungi Leucocalocybe mongolica
Source: J Fungi (Basel). 2022 Jun 28;8(7):680. doi: 10.3390/jof8070680 (PMC9324422; doi:10.3390/jof8070680)
Supplement: Supplementary file 1 [file jof-08-00680-s001.zip › Supplementary Material File S1.pdf]

Supplementary Material File S1 : Method of soil chemistry properties assess

1. NPK

1.1. N

The content of total nitrogen in soil was determined by Kjeldahl method with sulfuric acid-accelerator digestion [1].

1.2. Nitrate-N

The contents of nitrate nitrogen in soil were determined by extraction with potassium chloride solution and spectrophotometry [2,3].

1.3. Nitrite-N

The contents of nitrite nitrogen in soil were determined by extraction with potassium chloride solution and spectrophotometry [2,3].

1.4. Alkeline-N

The content of soil alkali-hydrolyzed nitrogen was determined by alkali-hydrolyzed diffusion method [4].

1.5. Ammonia-N

The contents of ammonium nitrogen in soil were determined by extraction with potassium chloride solution and spectrophotometry [2,3].

1.6. N

The content of total nitrogen in soil was determined by Kjeldahl method with sulfuric acid-accelerator digestion

1.7. Micro-N

The contents of Micro-nitrogen were determined by chloroform fumigation [5]

1.8. P

The content of total phosphorus in soil was determined by NaOH alkali melting and molybdenum-antimony resistance spectrophotometry. [6]

1.9. Organo-P

The content of organic phosphorus in soil was determined by burning method [7].

1.10. Inorganic-P

The content of inorganic phosphorus in soil was determined by mo-Sb resistance colorimetry with 1mol/L hydrochloric acid extraction [8].

1.11. Alkaline-Available P

The content of alkaline available phosphorus in soil was determined by molybdenum-antimony resistance colorimetric method using ammonium fluorine-hydrochloric acid solution and sodium bicarbonate solution [9].

1.12. K

The content of total potassium in soil was determined by NaOH alkali melting and flame photometer. [1]

1.13. Available-K

The content of soil available potassium was determined by ammonium acetate extraction and flame photometer [10].

1.14. Slowly Available-K

The content of slow available potassium in soil was determined by hot nitric acid extraction and flame photometer [10].

2. Soil Enzyme Activity

2.1. Cellulase

The contents of cellulase in soil were determined by colorimetric method of 3, 5-dinitrosalicylic acid [11,12].

2.2. Urease

Soil urease activity was measured by Micro Soil Urease(UE) Assay Kit (BC0125-100T/48S, Solarbio, Beijing, China).

2.3. Sucrase

The contents of sucrase in soil were determined by colorimetric method of 3, 5-dinitrosalicylic acid [11,12].

2.4. Catalase

The content of catalase in soil was determined by potassium permanganate titration [11,12].

2.5. Dehydrogenase

Soil dehydrogenase content was determined by TTC (2, 3, 5-triphenyltetrazole chloride) colorimetry [11,12].

2.6. β-glucosidase

Determination of β -glucosidase content in soil by p-nitrophenol colorimetric method [13].

2.7. Acid Phosphatase

The contents of acid phosphatase in soil were determined by phenyldisodium phosphate colorimetric method [11,12].

2.8. Neutral Phosphatase

The contents of neutral phosphatase in soil were determined by phenyldisodium phosphate colorimetric method [11,12].

2.9. Alkaline Phosphatase

The contents alkaline phosphatase in soil were determined by phenyldisodium phosphate colorimetric method. [11,12]

3. Other Soil Properties

3.1. Organic Matter

The contents of soil organic matter were determined by potassium dichromate oxidation-external heating method [14].

3.2. Carbon

The total carbon content of soil was determined by combustion oxidation - nondispersive infrared spectroscopy [15].

3.3. Dissolved organic carbon

The content of soil dissolved organic carbon was determined by carbon and nitrogen analyzer [16].

3.4. Micro-Carbon

The contents of Micro-carbon were determined by chloroform fumigation [5].

3.5. Boron

The content of total boron in soil was determined by ICP-OES after melting with naOH. [17]
ICP-OES: Inductively Coupled Plasma Optical Emission Spectrometer.

3.6. Available Boron

The available boron content in soil was determined by boiling water extraction ICP-OES method [18].
ICP-OES: Inductively Coupled Plasma Optical Emission Spectrometer

3.7. Sulfur

Total sulfur content in soil was determined by ICP-OES with nitric acid-perchloric acid digestion [19].
ICP-OES: Inductively Coupled Plasma Optical Emission Spectrometer

3.8. Available Sulfur

The soil was extracted with Ca(H2PO4) 2-HOAC and the soil available sulfur content was determined by barium sulfate turbidimetric method [20].

3.9. Salt

The total content of exchangeable salts in soil was determined by ammonium acetate exchange - neutralization titration [3].

3.10. Mn

The contents of available manganese in soil were determined by DTPA-TEA extraction method [21].
DTPA: Diethylenetriaminepentaacetic Acid Pentasodium Salt
TEA: Triethanolamine

3.11. Fe

The contents of available iron in soil were determined by DTPA-TEA extraction method [21].
DTPA: Diethylenetriaminepentaacetic Acid Pentasodium Salt
TEA: Triethanolamine

3.12. Cu

The contents of available copper in soil were determined by DTPA-TEA extraction method [21].
DTPA: Diethylenetriaminepentaacetic Acid Pentasodium Salt
TEA: Triethanolamine

3.13. Zn

The contents of available zinc in soil were determined by DTPA-TEA extraction method [21].
DTPA: Diethylenetriaminepentaacetic Acid Pentasodium Salt
TEA: Triethanolamine

3.14. Na

The contents of sodium in soil were determined by ICP-OES after deionized water extraction [7].
ICP-OES: Inductively Coupled Plasma Optical Emission Spectrometer

3.15. Mg

The contents of magnesium in soil were determined by ICP-OES after deionized water extraction [7].
ICP-OES: Inductively Coupled Plasma Optical Emission Spectrometer

3.16. Ca

The contents of calcium in soil were determined by ICP-OES after deionized water extraction [7].
ICP-OES: Inductively Coupled Plasma Optical Emission Spectrometer

References

1. Method for determination of total potassium in soils. PRC National Standard: 1988; Vol. NY/T 87-1988.
2. Determination of nitrate nitrogen in soil -- Ultraviolet spectrophotometry method. PRC National Standard: 2016; Vol. GB/T 32737-2016.
3. Lu, R. *Methods for soil agrochemical analysis*; China Agricultural Science and Technology Press: 2000.
4. Nitrogen determination methods of forest soils. PRC National Standard: 2015; Vol. LY/T 1228-2015.
5. Society, A.C.P.C.o.C.S. *Methods for conventional analysis of soil agrochemistry*; Science Press: 1983.
6. Method for etermination of soil total phosphorus. PRC National Standard: 1988; Vol. NY/T 88-1988.
7. Bao, S. *Soil agrochemical Analysis (3rd edition)*; China Agriculture Press: 2000.
8. González Medeiros, J.J.; Pérez Cid, B.; Fernández Gómez, E. Analytical phosphorus fractionation in sewage sludge and sediment samples. *Analytical and bioanalytical chemistry* **2005**, *381*, 873-878, doi:10.1007/s00216-004-2989-z.
9. Soil Testing – Part7:Method for determination of avalible phosphorus in soil. PRC National Standard: 2014; Vol. NY/T 1121.7-2014.
10. Determination of exchangeable potassium and non-exchangeable potassium content in soil. PRC National Standard: 2004; Vol. NY/T 889-2004.
11. Lin, X. *Principles and methods of soil microbial research*; Higher Education Press: 2010.
12. Li, Z.; Luo, Y.; Teng, Y. *Soil and environmental microbiology methods*; Science Press: 2008; pp. 395-402.
13. Eivazi, F.; Tabatabai, M.A. Glucosidases and galactosidases in soils. *Soil Biology and Biochemistry* **1988**, *20*, 601-606, doi:10.1016/0038-0717(88)90141-1.
14. Soil testing. Part 6: Method for determination of soil organic matter. PRC National Standard: 2006; Vol. NY/T 1121.6-2006.
15. Soil. Determination of organic carbon. Combustion oxidation nondispersive infrared absorption method. PRC National Standard: 2014; Vol. LY/T 1228-2015.
16. Li, T.; Guo, Z.; Kou, C.; Lu, J.; Zhang, x.; Yang, X. Effects of extraction methods on determination of soil soluble organic carbon. *Ecology and Environmental Sciences* **2017**, *26*, 1878-1883, doi:10.16258/j.cnki.1674-5906.2017.11.008.
17. Li, D.; Zhang, X. Direct determination of fourteen elements in soil by ICP-AES and ISE. *Journal of Instrumental Analysis* **1993**, 66-69.
18. Soil Testing. Part 8: Method for determination of soil avalible boron. PRC National

Standard: 2006; Vol. LY/T 1228-2015.
19. Zhang, X.; Zheng, Y.; He, X.; Lin, C.; Chen, N. Improvement of extraction and determination method of soil total sulfur. *Journal of Anhui Agricultural Sciences* **2017**, *45*, 91-93, doi:10.13989/j.cnki.0517-6611.2017.17.032.
20. Soil Testing. Part 14: Method for determination of soil avalible sulphur. PRC National Standard: 2006; Vol. NY/T 1121.14-2006.
21. Determination of available zinc, manganese, iron, copper in soil. Extraction with buffered DTPA solution. PRC National Standard: 2004; Vol. NY/T 890-2004.
